# Mesenchymal Stem Cells Enhance Liver Regeneration via Improving Lipid Accumulation and Hippo Signaling

**DOI:** 10.1155/2018/7652359

**Published:** 2018-05-13

**Authors:** Yang Liu, Faji Yang, Jun Li, Jinglin Wang, Xun Wang, Yuheng Zhang, Xianwen Yuan, Wei Zhu, Xiaolei Shi

**Affiliations:** ^1^Department of Hepatobiliary Surgery, The Affiliated Drum Tower Hospital of Nanjing University Medical School, Nanjing, Jiangsu 210008, China; ^2^Department of Anesthesiology, The Affiliated Drum Tower Hospital of Nanjing University Medical School, Nanjing, Jiangsu 210008, China

## Abstract

The liver has the potential to regenerate after injury. It is a challenge to improve liver regeneration (LR) after liver resection in clinical practice. Bone morrow-derived mesenchymal stem cells (MSCs) have shown to have a role in various liver diseases. To explore the effects of MSCs on LR, we established a model of 70% partial hepatectomy (PHx). Results revealed that infusion of MSCs could improve LR through enhancing cell proliferation and cell growth during the first 2 days after PHx, and MSCs could also restore liver synthesis function. Infusion of MSCs also improved liver lipid accumulation partly via mechanistic target of rapamycin (mTOR) signaling and enhanced lipid *β*-oxidation support energy for LR. Rapamycin-induced inhibition of mTOR decreased liver lipid accumulation at 24 h after PHx, leading to impaired LR. And after infusion of MSCs, a proinflammatory environment formed in the liver, evidenced by increased expression of IL-6 and IL-1*β*, and thus the STAT3 and Hippo-YAP pathways were activated to improve cell proliferation. Our results demonstrated the function of MSCs on LR after PHx and provided new evidence for stem cell therapy of liver diseases.

## 1. Introduction

The liver is a central metabolic organ that is involved in synthesis, storage, and metabolism. It has a remarkable capacity to restore its loss of mass caused by injuries. Such regenerative capacity provides a safeguard for patients with liver diseases. Therefore, enhancing liver regeneration (LR) will benefit patients after clinical treatment, such as partial hepatectomy (PHx) [1]. Moreover, the 70% PHx model is the most well-established model to study the ability of LR. In this model, liver mass can be recovered in 7–10 days after PHx, especially in the first 2 days post operation, which is supported by hepatocellular hypertrophy (cell growth) and hyperplasia (cell proliferation) [2].

Multiple signaling pathways and mechanical events are activated after PHx to ensure successful LR, such as IL-6-STAT3, HGF/c-Met, and Wnt-*β*-catenin [3, 4]. Right after PHx, interleukin 6 (IL-6), tumor necrosis factor (TNF), and hepatocyte growth factor (HGF) are rapidly upregulated in blood and remaining liver, causing a robust regenerative response and triggering hepatocyte proliferation. IL-6 and TNF are proinflammatory cytokines; in recent studies, it is believed that there are links between inflammation and regeneration of injured tissues, especially for intestine and liver [5]. For instance, IL-6 activates yes-associated protein (YAP) and Notch through gp130, but independently of STAT3 in injured mucosal tissues, which induces epithelial regeneration in the intestine [6]. Moreover, a number of inflammatory signals (such as IL-6) play a major role in LR [7] but its underlying mechanism is still unclear.

As LR progresses, hepatocytes enter into a cell cycle and start mitosis in order to meet the hepatostat, a process which needs energy and materials for DNA and protein synthesis. Previous studies have confirmed that lipids, triglycerides (TG), fatty acids (FA), and cholesteryl esters can be accumulated in hepatocytes at the peak of hepatocyte proliferation for 2–3 days, which is called transient regeneration-associated steatosis (TRAS) [8]. Disruption of hepatic adipogenesis is associated with impaired LR [9, 10], which can provide energy through *β*-oxidation and is the material source of new cell membrane formation.

Although the liver has a strong regenerative ability, it is still a challenge for patients recovering after surgery. Recently, stem cell-based therapy has been approved as an alternative choice for various liver diseases, such as acute liver injury and liver fibrosis [11, 12]. Mesenchymal stem cells (MSCs) are multipotent stromal cells existing in many tissues [13]. Experiments have confirmed the therapeutic ability of MSCs for acute and chronic liver diseases based on self-renewing and differentiating ability and immunomodulatory characteristics [14]. Clinical trials have confirmed the curative effect of MSCs on liver diseases [15, 16]. Previous studies proved that, in the models of PHx, infusion of MSCs increased the expressions of HGF, IL-6, and IL-10 and activated the IL-6-STAT3 pathway, thus improving the ability of LR [17–19]. However, the mechanisms of MSCs in improving LR after PHx remain unclear.

In the present study, we aimed to explore the mechanism of MSCs for improving LR after PHx. We focused on the ability of MSCs to modulate lipid accumulation and inflammation-related regeneration in liver.

## 2. Materials and Methods

### 2.1. Isolation of MSCs

Bone marrow-derived MSCs were isolated from 4- to 6-week-old male Sprague-Dawley (SD) rats. Briefly, femurs and tibias were sterilely dissected from rats, and soft tissues were carefully removed. After washing with PBS, bone marrow cells were flushed by low-glucose Dulbecco's modified Eagle's medium (DMEM, Gibco, USA), then centrifuged at 1200 rpm for 5 min, and resuspended in DMEM supplemented with 10% fetal bovine serum, penicillin (100 U/mL), and streptomycin (100 mg/mL). Subsequently, isolated cells were cultured at 37°C in a humidified atmosphere containing 5% CO_2_. Nonadherent cells were removed after 24 h, and culture medium was refreshed every 3 days. MSCs used for experiments were in passages 3–6.

### 2.2. Experimental Design of Rat PHx

Male SD rats aged 6–8 weeks were purchased from the Laboratory Animal Center of the Affiliated Drum Tower Hospital of Nanjing University Medical School, China. All rats were housed with 12 h light/12 h dark cycles and fed with normal diet and water. Moreover, a 70% PHx model was established under anesthesia using isoflurane as previously reported [20]. Rats were randomly divided into three groups (*n* = 6 at each time point). The rats in the sham control group only underwent laparotomy, while the model of 70% PHx was established in the other two groups, in which the left and middle lobes of the liver were removed. One 70% PHx group was injected with 1 mL PBS via the tail vein, namely the PHx + PBS group, while another 70% PHx group was administered with 1 mL MSC suspension (1 × 10^6^) in PBS via the tail vein, namely the PHx + MSC group. Then, rats were sacrificed at 1, 2, and 7 days after PHx. The remaining liver was weighed after sacrificing, then tissues were snap frozen in liquid nitrogen and stored at −80°C for further analysis. For histological analysis, tissues were fixed in 10% formaldehyde and embedded in paraffin. In order to inhibit mechanistic target of rapamycin (mTOR) signaling, an mTOR inhibitor, rapamycin (Rap, Selleck, USA), was given at a dose of 1 mg/kg i.p. 12 h post-PHx and every 24 h thereafter.

### 2.3. Western Blotting

Whole liver protein was extracted from rat liver using lysis buffer (KeyGen Biotech, Nanjing, China) containing protease and phosphatase inhibitors. Protein concentration was determined by a BCA protein quantitation assay (KeyGen Biotech, Nanjing, China). Briefly, 20 *μ*g of proteins were subjected to 8%–12% SDS-PAGE and then transferred onto PVDF membranes. Blots were blocked with blocking buffer for 2 h and then incubated with appropriate primary antibodies at 4°C overnight. Antibodies against GAPDH, PCNA, AKT, p-AKT, Bcl2, Bcl-xl, STAT3, p-STAT3, FASN, and Cyclin D1 were purchased from Abcam. Antibodies against mTOR, p-mTOR, 4EBP1, p-4EBP1, GSK3*β*, p-GSK3*β*, FOXO1, YAP, p70 S6K, p-p70 S6K, and SOCS3 were purchased from Cell Signaling Technology. Antibodies against *β*-catenin and SREBP1c were purchased from Santa Cruz Biotechnologies. After washing 3 times with Tris-buffered saline containing 1% Tween 20 (TBST), the membranes were incubated with the secondary antibody goat anti-rabbit IgG and goat anti-mouse IgG (Abcam, UK) at room temperature for 2 hours. After washing 3 times with TBST, signals of the target protein were developed using the enhanced chemiluminescence substrates (ECL, Thermo Fisher Scientific).

### 2.4. Histological and Immunohistochemical Analyses

Liver tissues were fixed in 4% paraformaldehyde and embedded in paraffin. Briefly, 5 *μ*m of paraffin-embedded liver sections were stained with hematoxylin and eosin (H&E) for morphological examination or stained with ki-67 (Abcam, UK) and *β*-catenin for routine immunofluorescence staining. The number of ki-67-positive cells was counted by Image-Pro Plus software in four randomly selected samples. For the visualization of hepatic lipid content, 8 *μ*m frozen sections were rehydrated and deposition of lipid droplets was detected by Oil Red O staining.

### 2.5. Measurement of Cell Size

Digital images of thin liver sections were prepared from formalin-fixed and paraffin-embedded samples stained by an immunohistochemical for *β*-catenin (Santa Cruz Biotechnology) to stain the hepatocyte membrane. Hepatocyte size was measured as area in pixels using ImageJ software in 20 hepatocytes per rat by an observer who was blinded to the treatment groups.

### 2.6. Real-Time Quantitative Polymerase Chain Reaction (RT-qPCR)

Total RNA was extracted from liver tissues using the TRIzol reagent (Invitrogen) according to the manufacturer's instructions. Purified RNA was reversely transcribed into cDNA using PrimeScript™ RT Master Mix (Takara, Japan) and then quantified by SYBR *Premix Ex Taq* (Takara, Japan) on an Applied Biosystems 7500 Fast Real-Time PCR System (Life Technologies). Data were analyzed using the 2^−ΔΔCT^ method. The primer sequences used were listed in Table S1.

### 2.7. Enzyme Linked Immunosorbent Assay (ELISA)

The plasma from each group of rats was obtained at 24 and 48 h after PHx, and the IL-6 concentration in plasma was measured by ELISA kits (eBioscience, USA).

### 2.8. Assessment of Liver Function

The plasma from rats was obtained at 1, 2, and 7 days after PHx, and levels of alanine transaminase (ALT), aspartate transaminase (AST), albumin (Alb), and TG were determined by an automated biochemical analyzer at the Affiliated Drum Tower Hospital of Nanjing University Medical School.

### 2.9. Liver TG

The TG level in liver tissues was measured using a triglyceride assay kit (Nanjing Jiancheng Bioengineering Institute, Nanjing, China) according to the manufacturer's instructions.

### 2.10. Statistical Analysis

All data were analyzed with GraphPad Prism 7.0 and presented as means ± standard deviation (SD). Statistical comparisons among groups were conducted using an unpaired *t*-test. *p* < 0.05 was considered as statistically significant.

## 3. Results

### 3.1. Infusion of MSCs Enhances LR after PHx in Rats

**Figure 1:**
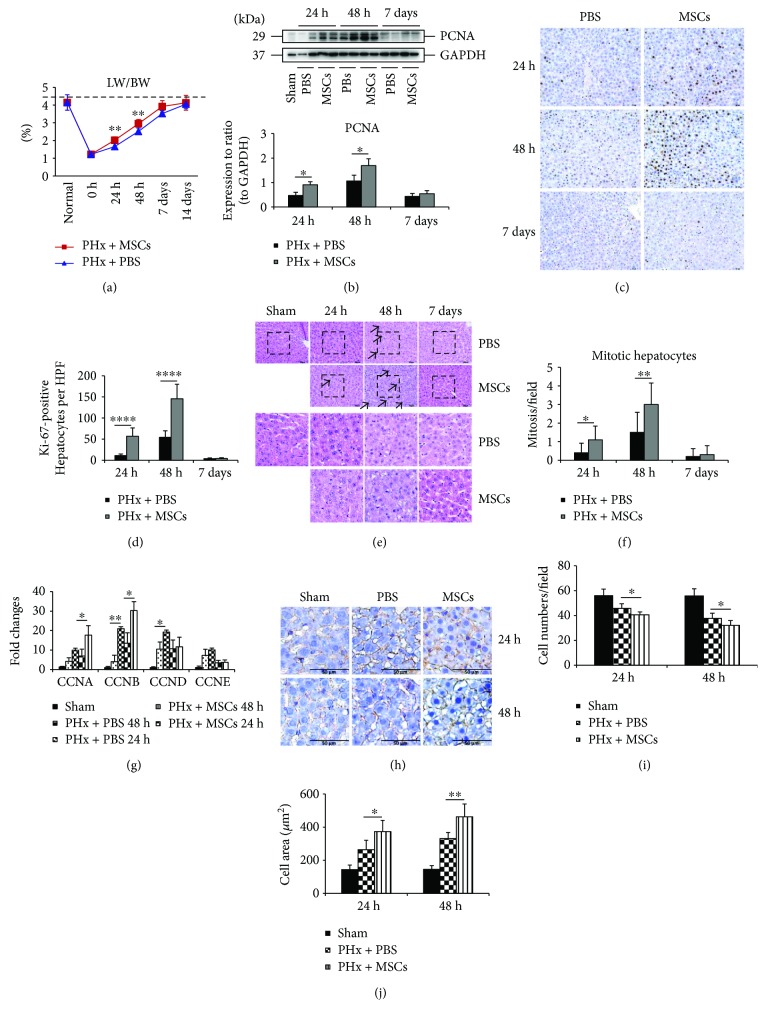
Effects of MSCs on LR after PHx. (a) The ratio of the remaining liver weight to total body weight (LW/BW) at 24 h, 48 h, and 7 days after PHx in rats (*n* = 6). (b) The expression of PCNA by Western blot in each group at 24 h, 48 h, and 7days after PHx. (c) Immunohistochemical staining of ki-67 in the liver specimens of each group at 24 h, 48 h, and 7 days after PHx. (d) Percentage of ki-67-positive cells in each group at 24 h, 48 h, and 7 days after PHx (*n* = 6). (e) RT-qPCR analysis showed the expression of genes related to mitosis (*CCNA*, *CCNB*, *CCND*, and *CCNE*) (*n* = 3). (f) & (g) H&E staining of liver specimens of each group at 24 h, 48 h, and 7 days after PHx and the number of mitotic hepatocytes on each group (*n* = 6). (h) Immunohistochemical staining of *β*-catenin in order to show the hepatocyte membrane and count the number of cells (*n* = 6). Values represent mean ± SD. ^∗^ *p* < 0.05; ^∗∗^ *p* < 0.01; ^∗∗∗∗^ *p* < 0.0001.

In the model of 70% PHx, infusion of MSCs enhanced LR as the ratio of liver weight to body weight (LW/BW) was significantly improved in the first 2 days (Figure 1(a)). However, such improvement was not detected at 7 days after PHx. Moreover, on day 7, the LW/BW ratio of both groups was almost restored to the same level as the normal group. And after 14 days, the LW/BW ratio was the same as the normal group. The expression of PCNA confirmed enhanced LR after infusion of MSCs (Figure 1(b)). The immunocytochemical staining of ki-67 showed that the number of ki-67-positive hepatocytes was greatly higher in the PHx + MSC group, especially at 48 h after PHx (Figures 1(c) and 1(d)). The H&E staining demonstrated that the number of mitotic hepatocytes was significantly increased in the PHx + MSC group at 24 h and 48 h (Figures 1(e) and 1(f)). In addition, the expressions of mitosis-related genes (*CCNA*, *CCNB*, *CCND*, and *CCNE*) were also increased in the PHx + MSC group (Figure 1(g)). These findings confirmed that infusion of MSCs could affect LR via promoting hepatocellular hyperplasia (hepatocyte proliferation). However, liver can also restore liver mass through hepatocellular hypertrophy (cell growth) [2]. Next, we investigated whether infusion of MSCs could improve LR via enlarging the cell size of hepatocytes. We stained the hepatocyte membrane using *β*-catenin antibodies and counted the number of cells per surface as previously described [21]. Figures 1(h) and 1(i) shows that the cell density was lower in the PHx + MSC group, and Figure 1(j) shows a larger cell size in the PHx + MSC group, which indicated that MSCs can enlarge hepatocyte cell size to restore liver mass after PHx. Taken together, infusion of MSCs could improve LR via hepatocellular hypertrophy and hyperplasia.

### 3.2. Infusion of MSCs Restores Liver Function after PHx

**Figure 2:**
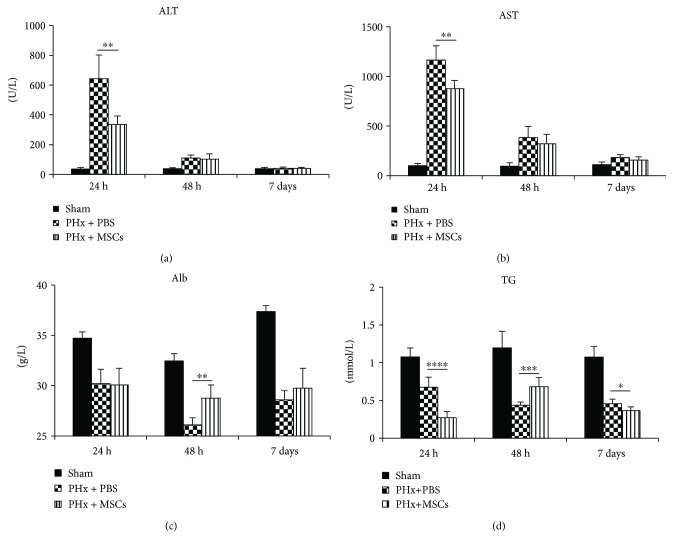
Serum levels of ALT, AST, Alb, and TG on each group at 24 h, 48 h, and 7 days after PHx. *n* = 6. Values represent mean ± SD. ^∗^ *p* < 0.05; ^∗∗^ *p* < 0.01; ^∗∗∗^ *p* < 0.001; ^∗∗∗∗^ *p* < 0.0001.

The liver is an organ with multiple functions, such as metabolism and synthesis. After LR, the levels of two serum biochemical parameters (ALT and AST), known to reflect hepatocyte damage, were increased after PHx, especially at 24 h (Figures 2(a) and 2(b)). However, as in Figure 1(e), there was no obvious liver damage like necrosis, suggesting that the liver was mildly damaged. But, infusion of MSCs significantly decreased the levels of ALT and AST (Figures 2(a) and 2(b)). Alb, associated with liver synthesis function, was also broken after PHx, obviously at 48 h, and infusion of MSCs could restore the Alb level (Figure 2(c)). Moreover, the level of serum TG was also influenced by infusion of MSCs (Figure 2(d)). At 1 week after PHx, levels of ALT and AST were recovered. These findings suggested that infusion of MSCs could partly decrease liver damage and recover liver synthesis function.

### 3.3. Infusion of MSCs Improves Liver Lipid Accumulation via mTOR Signaling

**Figure 3:**
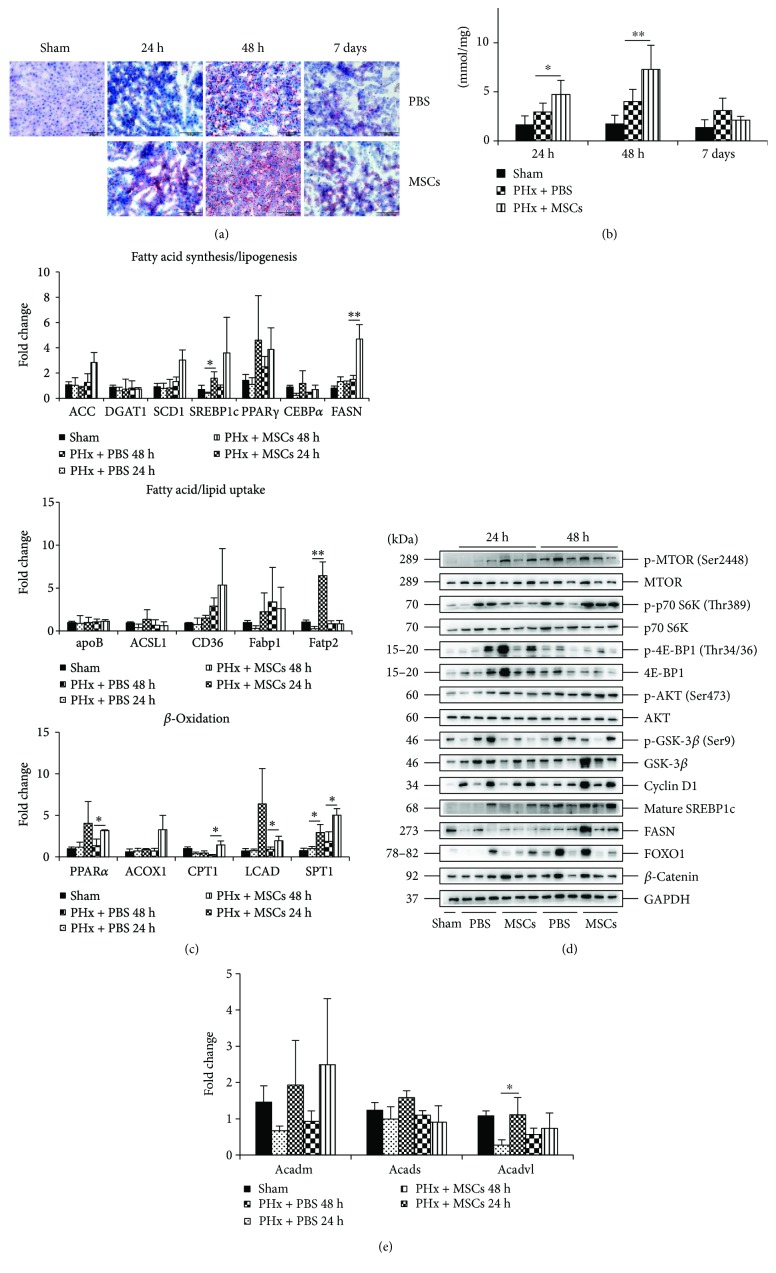
Effects of MSCs on lipid accumulation after PHx. (a) Oil Red O staining of liver specimens in each group at 24 h, 48 h, and 7 days after PHx. (b) The level of TG in liver specimens in each group at 24 h, 48 h, and 7 days after PHx (*n* = 6). (c) RT-qPCR analysis for the genes related to fatty acid synthesis and lipogenesis, fatty acid and lipid uptake, and *β*-oxidation (*n* = 3). (d) Western blotting of the mTOR signaling, its targets (Cyclin D1 and SREBP1c), and FOXO1 and FASN. (e) RT-qPCR analysis for the genes related to *β*-catenin-targeted *β*-oxidation. Values represent mean ± SD. ^∗^ *p* < 0.05; ^∗∗^ *p* < 0.01.

Figure 1(d) illustrates that the hepatocytes displayed steatotic change. Previous studies have shown that lipids play a great role in LR [8]. Therefore, we investigated the effect of MSCs on lipid accumulation after PHx. Oil Red O staining showed that lipid accumulation became more obvious in the PHx + MSC group (Figure 3(a)), and the level of TG in liver tissues was also higher in the PHx + MSC group (Figure 3(b)). These findings confirmed that infusion of MSCs could improve liver lipid accumulation. As in the liver, lipids can be accumulated via hepatocyte synthesis or uptake from blood. Next, we explored how MSCs improved the lipid accumulation in the liver. The expression of genes related to FA/lipid uptake (*apoB*, *ACSL1*, *CD36*, *Fabp1*, and *Fatp2*) and FA synthesis/lipogenesis (*ACC*, *DGAT1*, *SCD1*, *SREBP1c*, *PPARγ*, *CEBPα*, and *FASN*) showed that there was no significant difference between PHx + PBS groups and PHx + MSC groups (Figure 3(c)). This indicated that improved lipid accumulation after infusion of MSCs might not depend on FA/lipid uptake and FA synthesis/lipogenesis.

The mTOR is important for life, and it can regulate tissue growth, proliferation, and lipid synthesis [22]. mTOR promotes lipid synthesis through the sterol regulatory element-binding proteins (SREBPs), transcription factors controlling FA, and cholesterol biosynthesis [23], and the mRNA and protein level of *SREBP1c* was higher in the PHx + MSC group (Figures 3(c) and 3(d)). Thus we investigated the role of mTOR signaling in PHx after infusion of MSCs.  Figure 3(d) reveals that the expressions of mTOR signaling substrates, p-mTOR, p-S6K, p-AKT, and its target (Cyclin D1) were increased in the PHx + MSC group. In addition, the expressions of FA synthase (FASN), which is related to lipid homeostasis [24], were extremely higher in the PHx + MSC group at 48 h (Figure 3(d)). Therefore, infusion of MSCs could improve hepatic lipid accumulation via mTOR signaling after PHx, and further infusion could enhance lipid synthesis through FASN.

Lipids provide not only materials for cell membrane formation and expansion, but also energies for ATP synthesis, especially by *β*-oxidation in the liver [10]. Therefore, we investigated whether infusion of MSCs could also trigger lipid *β*-oxidation to provide energy for liver growth. The expressions of genes related to *β*-oxidation (*PPARα*, *CPT1*, *ACOX1*, *LCAD*, and *SPT1*) in the liver were also upregulated in the PHx + MSC group at 48 h (Figure 3(c)), indicating that infusion of MSCs could also trigger lipid *β*-oxidation. Previous studies have shown that *β*-catenin, a member of Wnt signaling, is a key factor for tissue growth and repair [25]. Moreover, *β*-catenin is an important protein for lipid catabolism in the liver [26]. Infusion of MSCs increased the expression of *β*-catenin (Figure 3(d)) after PHx at 24 h, but the expressions of *β*-catenin-related hepatic *β*-oxidation targets, acyl-COA dehydrogenases (*Acadvl*, *Acadm*, and *Acads*), were not significantly affected by MSCs (Figure 3(e)). Therefore, infusion of MSCs enhanced liver lipid accumulation via mTOR signaling and then improved *β*-oxidation to provide energy for LR. Moreover, infusion of MSCs only enhanced the expression of *β*-catenin and had no effect on the functions of *β*-catenin-related lipid catabolism.

### 3.4. Inhibiting mTOR by Rap Impairs LR after Infusion of MSCs

**Figure 4:**
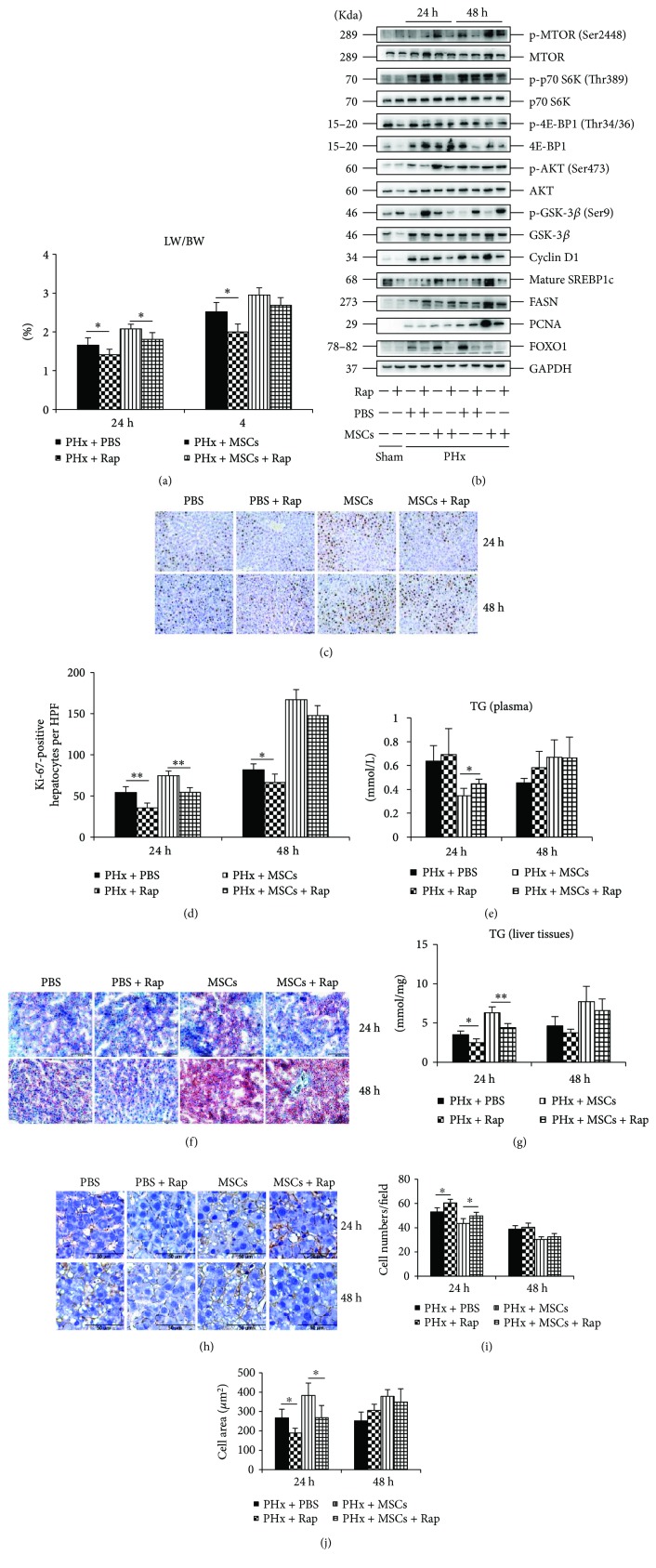
Effects of rapamycin on the infusion of MSCs in LR. (a) The ratio of the remaining liver weight to body weight (LW/BW) after Rap administration on each group at 24 h and 48 h after PHx (*n* = 6). (b) Western blotting analysis of mTOR signaling after rapamycin administration and the expression of the proliferation marker (PCNA). (c, d) ki-67 staining of liver specimens in each group and the number of ki-67-positive cells in each group (*n* = 4). (e) The level of TG in plasma (*n* = 4). (f) Oil Red O staining of liver specimens in each group at 24 h and 48 h. (g) The level of TG in liver specimens (*n* = 4). (h) *β*-Catenin staining of liver specimens (*n* = 4). Values represent mean ± SD. ^∗^ *p* < 0.05; ^∗∗^ *p* < 0.01.

To ensure the mTOR signaling in lipid accumulation after infusion of MSCs, we pretreated rats with Rap, an mTOR signaling inhibitor. After Rap administration, the LW/BW was significantly lower in both PHx + PBS and PHx + MSC groups, especially at 24 h after PHx (Figure 4(a)). However, there was no difference at 48 h between MSC groups and MSC + Rap groups, which indicated that there might be other compensatory mechanisms for LR. The ki-67 staining (Figures 4(c) and 4(d)) and expression of PCNA (Figure 4(b)) confirmed the delayed LR after Rap administration. The Oil Red O staining showed that lipid accumulation was inhibited by Rap administration at 24 h (Figure 4(f)), and the TG level in liver was also decreased by Rap administration at 24 h (Figure 4(g)). However, the level of plasma TG was not affected by Rap, except for the PHx + MSC group at 24 h after PHx (Figure 4(e)). There were no significant differences in Oil Red O staining and TG level in the liver at 48 h after Rap administration, which might be attributed to the expression of FASN (Figure 4(b)) and other lipid metabolism-related signals that were independent of mTOR signaling. Hepatocyte growth was inhibited by Rap in both PHx + PBS and PHx + MSC groups at 24 h after PHx (Figures 4(h)–4(j)), which might be attributed to the inhibited expression of p-S6K (Figure 4(b)), since a previous study has shown that S6K can improve cell growth [21]. Taken together, infusion of MSCs improved lipid accumulation to support LR via mTOR signaling at 24 h after PHx, while at 48 h, the improvement of LR might be independent of mTOR signaling after infusion of MSCs.

### 3.5. Infusion of MSCs Activates the IL-6-STAT3 and YAP Pathways

**Figure 5:**
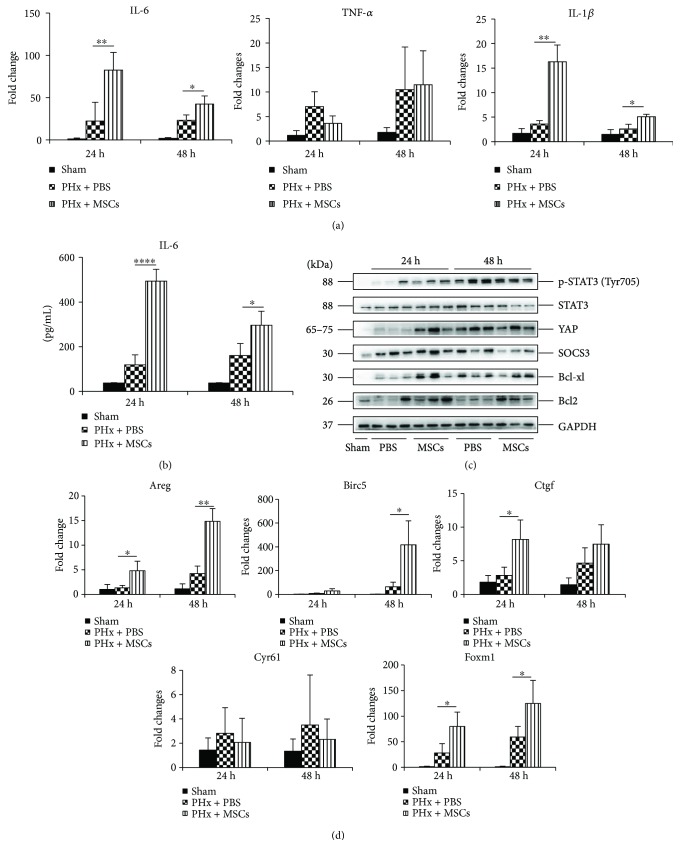
Effects of MSCs on cytokines and the STAT3 and Hippo pathways after PHx. (a) RT-qPCR analysis for the mRNA levels of IL-6, TNF-*α*, and IL-1*β* in the liver in each group at 24 h and 48 h after PHx (*n* = 3). (b) ELISA analysis for the serum levels of IL-6 in each group at 24 h and 48 h after PHx (*n* = 4). (c) Western blotting of the markers of STAT3 signaling (p-STAT3, STAT3, and SOCS3), antiapoptosis (Bcl2 and Bcl-xl), and YAP in the liver in each group at 24 h and 48 h after PHx. (d) RT-qPCR analysis for the mRNA levels of YAP targeted genes (*Areg*, *Birc5*, *Ctgf*, *Cyr61*, and *Foxm1*) (*n* = 3). Values represent mean ± SD. ^∗^ *p* < 0.05; ^∗∗^ *p* < 0.01.

We showed that infusion of MSCs could improve hepatocyte proliferation. However, its underlying mechanism remained largely unexplored. Previous studies have shown that the expressions of many cytokines are increased after LR, such as IL-6, HGF, and TNF, leading to improved LR. Therefore, we explored whether infusion of MSCs could affect these cytokines.  Figure 5(a) shows that infusion of MSCs could increase the mRNA levels of IL-6 and IL-1*β* in the liver, while the mRNA levels of TNF-*α* remained stable. ELISA showed that the level of serum IL-6 was also increased in the PHx + MSC group, especially at 24 h (Figure 5(b)), and the same expression pattern of IL-6 was observed in the liver. Consistent with previous studies, we found that infusion of MSCs activated STAT3 signaling, exhibiting increased expression of p-STAT3 at 24 h (Figure 5(c)). Moreover, infusion of MSCs also exerted an antiapoptotic effect, showing enhanced expressions of Bcl2 and Bcl-xl at 24 h (Figure 5(c)). After LR, a proinflammatory environment formed in the liver and infusion of MSCs could enhance this environment, showing increased expressions of IL-6 and IL-1*β* (Figure 5(a)). In addition, a recent study has shown that IL-6 can improve the expression of YAP, a key transcriptional coactivator of tissue growth, which links the inflammation to tissue regeneration [6]. Furthermore, YAP, the core of the Hippo pathway, has been proved to play an important role in regulating organ size, cell fate, and carcinogenesis in the liver [27], and the expression of YAP-targeted *CCNB* was increased in the PHx + MSC group (Figure 1(g)). Therefore, we explored the function of YAP in LR. Western blotting showed that YAP was upregulated after PHx, and infusion of MSCs could enhance the expression of YAP, especially at 24 h (Figure 5(c)). The expressions of YAP-targeted genes, *Areg*, *Birc5*, *Ctgf*, and *Foxm1*, were increased after PHx, and infusion of MSCs further significantly increased the expressions of these genes, but there was no effect on the expression of *Cyr61* (Figure 5(d)). Taken together, these findings confirmed that infusion of MSCs could establish an inflammatory environment in the liver after PHx, leading to improved cell proliferation via IL-6-STAT3 and Hippo signaling. Meanwhile, infusion of MSCs could also inhibit apoptosis.

## 4. Discussion

In this study, we showed that infusion of MSCs could promote LR after PHx in a rat model. We also investigated the mechanism of MSCs on LR. The major findings of this study were as follows: (a) infusion of MSCs could enhance LR via hepatocellular hypertrophy and hyperplasia; (b) infusion of MSCs recovered the impaired liver function and attenuated hepatocyte apoptosis; (c) infusion of MSCs could improve hepatocyte proliferation via the IL-6-STAT3 pathway and the expression of YAP; and (d) infusion of MSCs improved lipid accumulation and cell growth via mTOR signaling, especially at 24 h, while it inhibited mTOR which could impair LR.

Transient steatosis is necessary for LR, as lipids are the resource for new cell membrane formation and biosynthesis and energy source for proliferation, which are important for the regeneration process [8]. Studies have shown that reduced liver TG content, decreased lipid droplet accumulation, and disrupted hepatic adipogenesis are associated with impaired LR [9, 28]. We found that steatotic hepatocytes were more obvious, and the TG level was higher in the liver in the PHx + MSC group. Infusion of MSCs could improve lipid accumulation in the liver partly via mTOR signaling. And inhibiting mTOR by rapamycin impaired LR on both groups via inhibiting both hepatocyte proliferation and growth, and lipid accumulation was less obvious on rapamycin groups, especially at 24 h after PHx. Moreover, the genes related to *β*-oxidation were increased after infusion of MSCs, which support the energy for LR. But *β*-catenin-associated lipid catabolism in liver was not influenced after infusion of MSCs. *β*-Catenin might contribute to hepatocyte proliferation not through triggering lipid *β*-oxidation in this model. Although lipid is important for LR, if liver has severe steatosis before PHx, LR may be impaired because hepatocyte injury is increased, Kupffer cell-mediated inflammatory responses are aggravated, and lipid peroxidation is increased, leading to elevated reactive oxygen species (ROS) and lipotoxic metabolites (ceramides and diacylglycerols), which can trigger inflammation and hepatic apoptosis after PHx [29, 30].

MSCs not only have differentiation potential, but also immunomodulatory ability. Most recent studies have focused on the immunomodulatory ability of MSCs. Interestingly, MSCs have been shown to inhibit inflammatory response. A previous study has shown that adipose-derived stem cells can reduce the serum levels of IL-6 and TNF-*α* [18]. However, we found that the IL-6 level was increased in both the liver and serum after infusion of MSCs in our study, especially at 24 h after PHx, and the IL-1*β* expression was also increased, while TNF-*α* remained unchanged. IL-6 has both proinflammatory and anti-inflammatory effects [31]. The anti-inflammatory effect is mediated by a membrane-bound IL-6 receptor (mIL-6R), which is dependent on the STAT3 pathway to modulate hepatocyte proliferation [32]. Moreover, the elevated expressions of IL-6 and IL-1*β* after infusion of MSCs formed an inflammatory microenvironment in the liver, which could improve the recruitment of MSCs to the liver. Recent insights have shed light on the relationship between inflammation and tissue repair [5]. The cytokines, such as TNF, IL-6, IL-22, and IL-17, can improve regeneration through activating AP-1, NF-*κ*B, STAT3, YAP, and Notch [5]. YAP is the core of the Hippo pathway regulating epithelial tissue growth. YAP primarily binds to the TEAD family of transcription factors to regulate genes associated with proliferation, antiapoptosis, stemness, epithelial-mesenchymal transition, and so on [33]. We showed that the expressions of YAP and YAP-regulated genes were increased after infusion of MSCs. Some studies have shown the relationship between the Hippo-YAP and mTOR pathway [34, 35]. YAP can enhance the PI3K-mTOR via suppressing the expression of PTEN to control cell growth and cell proliferation [36]. In a future study, we should further explore the effects of MSC infusion on these two pathways as well as the bridge linking these two pathways.

MSCs could secrete plenty of growth factors and immunosuppressive factors, such as HGF, TSG6 (TNF-*α* stimulated gene/protein 6), prostaglandin E_2_, IL-10, and TGF-*β* (transforming growth factor *β*), to modulate the microenvironment and immune cells to keep homeostasis and facilitate the proliferation ability, which is called cell “empowerment” [13]. In this study, we only showed that infusion of MSCs could activate the IL-6-STAT3 and YAP pathways as well as mTOR signaling to improve lipid accumulation, leading to improved LR. However, it remains unclear how MSCs activate these signaling pathways, which may be through factors or extracellular vesicles secreted by MSCs, such as microRNA [37].

## 5. Conclusions

In the present study, we showed the potential of MSCs to improve LR through cell growth and cell proliferation. Moreover, infusion of MSCs could affect lipid accumulation in the liver. These data indicated that MSC transplantation might be an adjunctive method to recover liver function for patients with liver resection.

## Supplementary Materials

Supplementary Materials Table S1: the primer sequences used in this manuscript are shown.
