# Supplementary material for: Mesenchymal Stem Cells Enhance Liver Regeneration via Improving Lipid Accumulation and Hippo Signaling
Source: Stem Cells Int. 2018 May 13;2018:7652359. doi: 10.1155/2018/7652359 (PMC5971352; doi:10.1155/2018/7652359)
Supplement: Supplementary Materials — Table S1: the primer sequences used in this manuscript are shown. [file 7652359.f1.pdf]

Table S1. Sequence of primers used in experiments

Gene	Forward sequence	Reverse sequence
GAPDH	TTCAACGGCACAGTCAAG	TACTCAGCACCAGCATCA
CCNA	ATCTGACCGTTCCAACCA	GGCAAGGCACAATCTCAT
CCNB	CTTACACCAAGCACCAGAT	GTCCATTCACCGTTGTCA
CCND	ACTTCCTCTCCTGCTACC	GCTTCTTCCTCCACTTCC
CCNE	CTGGATGTTGGCTGCTTA	CACTGATAACCTGAGACCTT
IL-6	CAGAGTCATTCAGAGCAATAC	GATGGTCTTGGTCCTTAGC
TNF-α	TGTTCATCCGTTCTCTACC	CCACTACTTCAGCGTCTC
IL-1β	CAGAACATAAGCCAACAAGT	ACACAGGACAGGTATAGATTC
HGF	TCATTGGTAAAGGAGGCA	GTCACAGACTTCGTAGCGTA
IL-10	AGCCAGACCCACATGCTCCGA	ACAGGGGAGAAATCGATGACA GCGT
Areg	GCGGAACCAATGAGAACT	TGAGTCGTCCACAATATAGC
Birc5	ACAGGAGACCGTGACATT	CATCAGAAGGACAGAAGAAGA
Cyr61	CGAAGATGGCGAGATGTT	GATGAGCAAGGCACTGTT
Ctgf	CTATGATGCGAGCCAACT	CGGTAGGTCTTCACACTG
Foxm1	TTGAGGTAGAGGCAGGAG	GATAGAGGTGGTAATGAGACA
ACC	GGACAACACCTGTGTGGTAGAA	CGTGGGGATGTTCCCTCT
DGAT1	TGCTCTGGCATCATACTCCA	TACAGTGTTCTGGGCAGCAG
SCD1	TCCCCTCCTCCAAGGTCTAC	GCTCCACAAGCGATGAGC
SREBP1c	GCTACCGTTCCTCTATCAAT	GTTGCTGTGCTGTAAGAAG
PPARγ	TTGAATGACCAAGTGACTCT	CGTGCTCTGTGACAATCT
CEBPα	GCTCCTCCATCTACATTCC	ACCACTTGCTTAACCACAT
FASN	GGCTTGGTGAACTGTCTC	GGCTTGTCCTGCTCTAAC
apoB	ACAACATACGACAGCAAGA	AGGTGGAAGTGACAGACA
ACSL1	TGGGCACAGAAGAGAGGATT	ATGGCTTCAAACCAGCATTC
CD36	AATGAGACTGGGACCATCG	CTCCAACACCAAGTAAGACCA T

Fabp1	AAATCGTGCATGAAGGGAAG	GTCTCCAGTTGGCACTCCTC
Fatp2	AGTACATCGGTGAACTGCTTCGGT	CAATGTTGCCTTCAGTGGAAGCGT
PPARα	GAAGCCTACCTGAAGAACTT	GAGGACAGCATCGTGAAG
ACOX1	CTCACTCGAAGCCAGCGTTA	TTGAGGCCAACAGGTTCCAC
CPT1	CGGTTCAAGAATGGCATCATC	TCACACCCACCACCACGAT
LCAD	CCCTGGTTTCAGCCTCCATT	TCACTCCCAGACCTTTTGGC
SPT1	ACCTGGAGCGACTGCTAAAA	ATCCCATAGTGCTCGGTGAC
Acadm	AGAGCAGGAAGGCATCAT	CAGCAATAATCACAGGCATT
Acads	GTCTGTGGAACTACCTGAG	CTTCTTCACCTGCGATGT
Acadvl	TCTCCTCTGATGCTTCCA	CTCCTCCACCTTCTCCAA
